# Association of stress management behavior and diabetic self-care practice among diabetes type II patients in North Shoa Zone: a cross-sectional study

**DOI:** 10.1186/s12913-023-09752-6

**Published:** 2023-07-19

**Authors:** Akine Eshete, Sadat Mohammed, Tilahun Deresse, Tewodros Kifleyohans, Yibeltal Assefa

**Affiliations:** 1 Department of Public Health, Debre Berhan University, Debre Berhan, Ethiopia; 2 School of Medicine, Debre Berhan University, Debre Berhan, Ethiopia; 3 School of Public Health, the University of Queensland, Brisbane, Australia

**Keywords:** Diabetic Self-care Practice, Stress management behavior, Patients with type II diabetes, North Shoa Zone

## Abstract

**Background:**

The cornerstone of diabetes management is the self-care behavior of diabetics. However, many people with diabetes do not fully engage in these activities. Effective stress management behaviors have a positive impact on diabetes self-care. The purpose of this study was to investigate the relationship between self-care behaviors in people with diabetes and stress coping behaviors in people with type II diabetes.

**Method:**

A facility-based cross-sectional study was undertaken in the North Shoa zone from March 2 to 29, 2022. The study involved 432 types II diabetic patients who were chosen at random from eight public hospitals. Eight item stress coping techniques tools was used to measure stress management behavior. Data were entered into Epi Data V.3.1 and analyzed using SPSS version 22. Data for continuous variables were reported as means and standard deviations and percentages for categorical variables. Descriptive statistic was used to summarize study variables. Binary logistic regression models were used to assess associations between sociodemographic variables, stress-coping behaviors, and self-care behaviors. Binary logistic regression model was used investigate the association between diabetic self-care behaviors and stress-coping behaviors. A p-value ≤ 0.05 and an OR with a 95% CI are considered statistically significant associations.

**Result:**

the study showed that stress management behavior was observed in more than half of the patients (51.2; 95% CI; (46.5, 55.6). The study found that stress management behavior was associated with diabetic self-care practice (X2, 17.7; p < 0.0001). Patients with good stress management behavior (AOR = 2.0, 95% CI = (1.3, 3.0)), good perception (AOR = 2.3, 95% CI = (1.5, 3.4)), and family support (AOR = 2.3, 95% CI = (1.5, 3.6)) were more likely to conduct diabetes self-care.

**Conclusion:**

This study shows that stress management behaviors and coping techniques are associated with self-care behavior and lead to significant improvements in diabetes self-care practices. Stress management and coping skills should be included in current systems as a common therapeutic service/treatment. Diabetes care practitioners should consider these factors when discussing diabetes self-management during consultations.

## Background

Diabetes is a growing public health problem worldwide in the 21st century [1], especially type II diabetes (T2DM), which accounts for 90% of all diabetes cases [2]. Ethiopia has the highest prevalence of diabetes, ranging from 2.0 to 6.5% [3]. This rapid increase in diabetes requires self-management behaviors, especially in areas with inadequate medical care [4, 5]. People with good diabetes self-management have a positive impact on glycemic control [6].The goal of diabetes treatment is to maintain a better quality of life. Nevertheless, in many countries, including Ethiopia, diabetes self-care remains the most challenging aspect of diabetes care and management [7–11].

Stress management behaviors are skills that help individuals cope successfully with the demands and challenges of life [12]. Mental illness are known to exacerbate diabetes in many people and lead to poor management [13, 14]. People who do not have proper stress management skills may not behave appropriately. Studies have shown that stressful life events can make diabetes treatment and management less effective [15, 16]. Stressful life events in patients can negatively affect problem-solving skills and lead to poor self-management behavior [17]. Therefore, adaptive coping strategies are important for people with diabetes.

There is evidence that diabetics experiencing stress and depression have negative effects on self-care behaviors, affecting health status [18, 19]. Self-managing behavior requires problem-solving skills at all levels. There is evidence that diabetes management is significantly associated with perceived stress and problem-focused coping styles [20].

Applying stress management techniques is effective in improving diabetes management and behavioral control [15, 21]. Stress management plays an important role in the self-care of chronically ill patients [22]. Emotional support is associated with better diabetes self-care, and people with diabetes need help finding the optimal adaptive strategies to improve their quality of life.

Applying stress management techniques is effective in improving diabetes management and behavioral control [15, 21]. Stress management plays an important role in self-care for chronically ill patients [22]. Emotional support is associated with better diabetes self-care, and people with diabetes need help finding the optimal adaptive strategies to improve their quality of life [23]. Additionally, managing diabetic stress is important for people with diabetes as it can lead to improved self-care.

Stress management techniques should be integrated into diabetes care and delivered at all levels of the healthcare system. There is evidence that stress management programs promote stress management strategies and self-efficacy [24]. To our knowledge, no studies have investigated the association between stress management behaviors and diabetes self-care practices in Ethiopia. Therefore, the aim of this study was to examine the association between stress management behaviors and self-care practices. In addition, this study provides relevant information for evidence-based decision-making and design of appropriate community interventions, as well as planning and design of future behavioral promotion strategies and interventions. Additionally, the results of this study will help people with diabetes and their healthcare providers plan appropriate interventions to ensure optimal health.

## Method of the study

### Study design and area

The facility-based cross-sectional study design was employed at public hospitals in the North Shoa Zone from March 2 to 29, 2022. North Shoa is one of the thirteenth zones of the Amhara region located in northern Ethiopia. There are 24 districts, 3 municipalities, and 13 hospitals. All public hospitals have diabetes care and follow-up services.

### Subjects and sample selection

The sample size was determined using single population proportion formula that considers a proportion of self-care practice of 51.12% in Ethiopia in 2021 [10] with a level of precision of 5%, and 15% contingency for non-response, resulting in 442 people. This study included consenting patients between the ages of 20 and 70, while patients those who were unable to participate in the study based on physician judgment (e.g., due acute illness, mental illness, dementia) and patients with severe visual impairment were excluded from the study.

Eight out of 13 hospitals were randomly selected to participate in the study. Sampling frames were created for each selected hospital using registration log book. Study participants were recruited after being proportionally allocated to each hospital. Study participants were selected using a simple random sampling method. Since each patient had at least one appointment within a month, we waited up to a month for a selected study participant.

### Data collection method

A total of four nursing bachelor data collectors participated in the data collection process. Data collectors and supervisors are trained in the data collection process, including research objectives, questionnaire content, and maintaining confidentiality and privacy during data collection. All authors and supervisors are checked daily for completeness, accuracy, and consistency of the data. The questionnaire was tested for content validity and reliability. To validate the content of the tool, all survey questions were reviewed by two public health experts and physician from Debre Berhan University. Questions were evaluated for readability, understandability and content validity and recommendations were made. Each collected questionnaire was checked on daily basis for completeness. Additionally, the internal consistency of the tool was checked using the Cronbach alpha test for self-care and stress coping behaviors in diabetes. Thus, the reliability of the diabetes self-care behavior tool (Cronbach’s alpha = 0.88) and stress management behavioral tool (Cronbach’s alpha = 0.82) was calculated.

### Measurement of the outcome variables

The main outcome of this study was diabetic self-care practice. Diabetes self-care practices are measured using the Diabetes Self-Care Activity Summary tool, which includes four areas: diet, foot care, exercise, and blood glucose self-monitoring [25, 26]. Respondents marked the number of days the specified behavior occurred on an 8-point Likert scale (ranging from 0 to 7 days). An overall average score was calculated and the above averages indicate better self-care practices in people with diabetes.

Stress coping behavior was measured using eight items adapted from stress coping techniques and tools [27, 28]. An overall average score is calculated, with scores above the average indicating better stress management behaviors.

### Data management and analysis

Data were entered into Epi Data version 3.1 and exported to Statistical Package for Social Sciences (SPSS) version 22. Descriptive analysis was used to describe the frequency distribution of each variable in the study. Continuous variables are presented as mean ± standard deviation and categorical variables are presented as percentages. Associations between independent and outcome variables were analyzed using a binary logistic regression model. To avoid too many variables and unstable estimates in subsequent models, only variables reaching p-values ​​less than 0.20 in bivariate analysis are kept in subsequent model analyses. P-values ​​ and 95% confidence intervals (CI) were used in judging the significance of the associations.

## Results

### Socio-demographic characters of the respondents

The study enrolled 432 people with type 2 diabetes and had a response rate of 98%. The average age of the respondents was 52 ± 11.1 SD years. More than half of the respondents (52.1%) were male. The majority of respondents 346 (80.1%) were Orthodox Christians, 257 (62%) were urban dwellers, and 324 (75%) were married. Additionally, the majority of participants received formal education. Of all respondents, half (50%) of their household income is classified as high income (Table 1).

**Table 1:** Socio-demographic characters of participants in North Shoa Zone, 2022

Variables	Frequency (%)
**Age of the respondent (Mean** **±** **SD; 52** **±** **11.1**	
Young age group (15–47 years)	130 (30.1)
Middle age group (48–63 years)	231 (53.5)
Elder age group (≥ 64)	71 (16.4)
**Sex of the respondent**	
Male	225 (52.1)
Female	207(47.9)
**The religion of the respondent**	
Orthodox	346 (80.1)
Muslim	25 (5.8)
Protestant	61(14.1)
**The residential area of respondents**	
Urban	257 (62.0)
Rural	175 (40.5)
**Current marital status of the respondent**	
Married	324 (75.0)
Single	30 (6.9)
Divorce	43 (10.0)
Widowed	35 (8.1)
**Educational status of the respondent**	
No formal education	189 (43.8)
Attending formal education	243 (56.2)
**Employment status of the respondents**	
Government employee	94 (21.8)
Merchant /Business /	79 (18.3)
Housewife	124 (28.7)
Farmer	135 (31.3)
**Family monthly Income (Ethiopia Birr)**	
1^ST^ Quartile (low-income level)	108(25.2)
2nd Quartile (middle-income level)	107(24.8)
3rd Quartile (high-income level)	216(50.0)

### Patient stress management behavior

Overall, more than half of the patients demonstrated stress management behaviors, 221 (51.2), 95% CI. (46.5, 55.6). As shown in Table 2, the most common reported actions taken by patients were take some time for relaxation each day (43.1%) and use specific methods to control my stress (42.4%) as sometimes and getting adequate sleep (41.2%) routinely (Table 2).

**Table 2:** Patient stress management behavior in North Shoa Zone, 2022

Item /variables (n = 432)	Frequency (n= %)			
	Never	Sometimes	Often	Routinely
Get enough sleep	30(6.9)	116(26.9)	108 (25.0)	178(41.2)
Take some time for relaxation each day	112 (25.9)	186 (43.1)	100 (23.1)	34(7.9)
Accept those things in my life which I cannot change.	146 (33.8)	159 (36.8)	96 (22.2)	31(7.2)
Concentrate on pleasant thoughts at bedtime.	138 (31.9)	154(35.6)	122 (28.2)	18(4.2)
Use specific methods to control my stress.	131(30.3)	183 (42.4)	93 (21.5)	25(5.8)
Balance time between work and play.	155 (35.9)	172 (39.8)	86 (19.9)	19(4.4)
Practice relaxation for 15–20 min daily.	170 (39.4)	174 (40.3)	68 (15.7)	20(4.6)
Pace me to prevent tiredness	82 (19.0)	177 (41.0)	136 (31.5)	37(8.6)
**Stress management behavior** Mean score ± SD 17.3 ± 4.7				
Having poor stress management behavior	211 (48.8); 95% CI; (44.4, 53.5)			
Having good stress management behavior	221 (51.2); 95% CI; (46.5, 55.6)			

### Association between diabetic self-care practice and patient stress management behavior

In the study, the overall self-management behavior of patients with type 2 diabetes was 249 (57.6%); 95% CI; 53.0, 62.1). The mean diabetes self-management behavior score was 19.8 ± 3.8 SD. Chi-square test analysis showed that adequate sleep (p < 0.0001), daily relaxation time (p = 0.03), and balance time between work and play (p = 0.047) were significantly associated with self-care behaviors in diabetic patients ( Table 3).

**Table 3:** Association between diabetic self-care practice and patient stress management behavior in North Shoa Zone, 2022

Variables (n = 432)	X^2^ test	Df	p-value
Get enough sleep	48.9	3	**P < 0.001**
Take some time for relaxation each day	14.0	3	**0.030**
Accept those things in my life which I cannot change	7.1	3	0.068
Concentrate on pleasant thoughts at bedtime	7.0	3	0.069
Use specific methods to control my stress	1.6	3	0.665
Balance time between work and play	8.0	3	**0.047**
Practice relaxation for 15–20 min daily	1.2	3	0.761
Pace me to prevent tiredness	6.3	3	0.097

### Association of diabetic self-care practice with patient stress management behavior and background information

In bivariate analysis, those with better stress coping behavior (COR = 2.3, 95% CI = (1.6, 3.4)), those with good perception (COR = 2.3, 95% CI = (1.5, 3.4)), those who have family support (COR = 2.3, 95% CI = (1.5, 3.6)), being a city dweller (COR = 1.7, 95% CI = (1.1, 2.4)) and people aged 48–63 years (COR = 1.9, 95% CI = (1.1, 3.3)) are more likely to practice diabetics self-care practice (Table 4).

In the adjusted model, patients who practice good stress management were twice more likely to practice self-care than their counterparts (AOR = 2.0, 95% CI = (1.3, 3.0). In comparison to their counterparts, individuals with good perception (AOR = 2.3, 95% CI = (1.5, 3.4)) and those with family support (AOR = 2.3, 95% CI = (1.5, 3.6)) are more likely to perform diabetic self-care (Table 4).

**Table 4:** Logistic regression analysis of diabetic self-care practice among the participants in North Shoa Zone, 2022

Variables	Diabetic self-care practice		p-value	COR (95%CI)	AOR (95%CI)
	Poor diabetic self-care practice	Good diabetic self-care practice			
**Patient stress management behavior**					
Having poor SMB	111 (25.7)	100 (23.1)		1	
Having good SMB	72 (16.7)	149 (34.5)	P < 0.0001	2.3 (1.6,3.4)*	2.0 (1.3,3.0)**
**Patients’ perception of diabetes**					
Wrong perception	110 (25.5)	99 (22.9)		1	1
Good perception	73 (16.9)	150 (34,7)	P < 0.0001	2.28 (1.5,3.4)*	2.5 (1.6,3.7)**
**Family support**					
Yes	117(27.1)	200(46.3)	P < 0.0001	2.3 (1.5,3.6)*	2.3 (1.5,3.7)**
No	66 (15.3)	49 (11.3)		1	1

Adjusted variables are the sex of the respondent, family income level, the residence of respondent, and age of the respondent

## Discussion

The purpose of this study was to investigate the relationship between stress management behavior and diabetes self-care in the North Shao Zone. This study found that stress management behaviors were associated with diabetes self-care (X^2^, 17.7; p < 0.0001). Previous studies [14, 29], support this finding and suggest that adequate stress management improves self-care in people with diabetes. On the other hand, improving diabetes self-care habits can effectively reduce stress in people with type 2 diabetes [30]. Therefore, educational programs and usual care services as stress management techniques should be considered as usual therapeutic services.

In this study, stress management led to significant improvements in self-care practices. Patients with good stress management skills were twice more to exercise diabetic self-care. Diabetes self-care necessitates a high level of stress-coping skills as well as problem-solving ability. Therefore, stress-coping behaviors are important for patients with type 2 diabetes. Even if stress management activity had a positive effect on diabetic self-care, over half of the patients in this study (51.2%) demonstrated good stress-coping behavior. To enhance self-care behaviors and stress management, it is necessary to implement stress coping strategies and problem-solving skills.

The most common stress reduction measures used in this study were getting enough sleep, focusing on happy thoughts in bed, and relaxing daily after the activity. The adoption and implementation of different stress management approaches is a priority as stress management techniques improve self-care behaviors of diabetics [15, 21]. In this study, among the various stress management techniques, getting enough sleep (p = 0.001), taking daily relaxation (p = 0.03) and time balance between work and play (p = 0.047) was significantly associated with diabetes self-care practice.

In this current study, patients with good perceptions are more likely to practice diabetic self-care. One reason could be that when patients have good insight, it can help them understand their health status and avoid confusion when taking diabetes self-care measures.

In this study, patients with good family support were more likely to have self-management behavior. Diabetes self-management behavior can be significantly improved with increased family support. Studies have shown that diabetes-specific supportive and family behaviors have a positive impact on individual self-management behaviors [31–33]. In addition, families are expected to assist and support patients’ self-management practices by assisting them in strategic planning, goal setting, and problem solving [34]. Therefore, to improve the health of adults with diabetes, it is important to support families who are committed to self-management of their diabetes. We need to build proper support and foster healthy relationships among all family members.

The current study has some limitations, including the possibility that self-reported measures may be biased in response and overestimate behavioral performance. This tool also needs more attention for accurate and reliable data.

### The implication of the study

Practicing stress management and coping skills is the preferred strategy for improving diabetes management behavior. This has been demonstrated in previous studies [15, 21, 22, 24] and in this study. Since stress management behaviors and coping skills are associated with diabetes self-management, diabetes professionals should consider these aspects when discussing diabetes self-management. In addition, the results indicate that stress management programs may have significant clinical benefits for patients with type II diabetes. Therefore, routine care and education programs should address diabetes self-care activities and coping skills that influence health-related behaviors and decision-making.

In summary, the following key program areas attract the attention of policymakers and service providers; (1) As a routine therapeutic or therapeutic service, stress management strategies and coping skills should be integrated into existing systems. (2) Create multiple stress management methods to reach large populations. (3) Pay special attention to the patient’s barriers, analyze them deeply and integrate them into their daily activities.

## Conclusion

The study results showed that stress management behavior significantly improved diabetes self-management behavior and had a positive association with it. Patients with good stress management behaviors are more likely to engage in diabetes self-management activities. In addition, patients with good awareness and patients with family support were more likely to have diabetes self-care.

## Data Availability

All data generated in this study are included in the manuscript. Datasets are available upon reasonable request from the corresponding author.

## Abbreviations

DM: Diabetes Mellitus
SPSS: Statistical Package for Social Sciences
SMB: Self-Management Behavior
T2DM: Type II Diabetes Mellitus
